# Young people are experts and leaders in the HIV response

**DOI:** 10.1002/jia2.25569

**Published:** 2020-08-31

**Authors:** Tinashe G Rufurwadzo, Audrey Inarukundo, Ikka Noviyanti, Miguel A Subero

**Affiliations:** ^1^ Global Network of Young People Living with HIV (Y+ Global) Harare Zimbabwe; ^2^ Réseau National des Jeunes Vivant avec le VIH (RNJ+) Bujumbura Burundi; ^3^ Youth LEAD Asia Pacific Network of Young Key Populations Bangkok Thailand; ^4^ Regional Network of Youth living with HIV Latin America and the Caribbean (J+LAC) México City Mexico

**Keywords:** adolescents, young people, HIV, sexual and reproductive health and rights, peer support, advocacy, leadership

It is 2020 and there are many examples of how young people are contributing to HIV prevention, treatment, support and care efforts globally. We do this as advocates, researchers, educators and service providers, among other roles.

This has not just happened overnight. We have had to push for meaningful leadership of young people in initiatives that affect our lives. In some contexts, we are still perceived as children who should stay quiet while adults and other experts say and do things for us. However, young people are increasingly taken seriously, as experts of our own lives, with clear ideas of what we are experiencing and need, as well as innovative ideas that respond to those needs.

Different factors have facilitated and supported our leadership, including organizations that believe in us and show it by funding and/or mentoring us, and programmes that enable us to be creative and deliver for our peers and for our communities. Processes and mechanisms placing young people at the table are critical to ensure that energies are spent taking up and making the most of these spaces.

There are a number of roles we play in the HIV response across advocacy, research, peer education and service delivery, using our experiences and skills to push change at community, national, regional and global levels.

## 1 YOUNG PEOPLE AS ADVOCATES

We are the most effective advocates for our own health and wellbeing and that of our peers. We know the barriers and solutions to accessing health services. Some of these solutions, such as comprehensive sexuality education, may not be popular in some contexts. However, we push the conversation, even when it is uncomfortable, because it is important that we speak to our realities in order to propel change.

Examples of young people playing an effective role as advocates can be found in the Resilient and Empowered Adolescents and Young people (READY) movement [1], which supports adolescents and young people in our diversity to understand our sexual and reproductive health rights and support our peers in making healthy choices. Most specifically, through the READY + project, and in line with research recommendations [2], networks of young people living with HIV in East and Southern Africa have been successfully advocating within parliamentary committees and technical working groups to lower the age of consent for HIV testing from 18 to 15 years. Elsewhere, READY advocates have brought the voices of young people to international conferences on HIV and to the UN to speak out on barriers to accessing healthcare [3].

## 2 YOUNG PEOPLE AS RESEARCHERS

There are a number of studies that research our behaviours and investigate health outcomes of specific interventions. However, we are not only subjects of research. We are also researchers, helping answer complex questions.

For example in 2019 young researchers led a trial to evaluate the effectiveness of the Zvandiri model for HIV‐related and psychosocial support services for adolescents living with HIV in rural districts of Zimbabwe [4]. The study found that adolescents receiving Zvandiri services and Ministry of Health and Child Care services were 42% more likely to be virologically suppressed than adolescents receiving Ministry of Health and Child Care services alone. Not only were young researchers involved in the rollout of the trial, they were also involved in taking up and using the findings to coordinate the successful implementation of the Zvandiri model within the intervention clinics at the rural district level.

## 3 YOUNG PEOPLE AS EDUCATORS

Since the HIV response began, young people have played a role as peer educators, passing on information to our peers through support groups, after school clubs and other settings. These efforts are ongoing, and the content of education and way young people educate their peers has evolved. Young people deliver sexuality education on sensitive topics such as same‐sex relationships, intergenerational relationships, violence and safe abortion to respond to the real questions and experiences of young people so they can stay healthy and make informed life and health choices [5].

For example Youth LEAD [6], a network of young key populations in Asia and the South Pacific, has a number of programmes that support peer education through 14 youth‐led organizations to share information they may have received at school or at home with their peers. This includes information about sexuality and sexual choices and behaviours. Knowledge is power – and that remains the case for young people who, like others, need to stay on top of research and developments, especially those on work towards achieving the sustainable development goals [7] in order to maximize our own health and care for peers.

## 4 YOUNG PEOPLE AS SERVICE PROVIDERS

Young people are also no longer only recipients of care. We are active in service provision both within health facilities and through outreach activities [8]. There are innovative collaborations between youth networks and organizations and health facilities to deliver quality care to young people – care that is non‐judgemental, competent and directly responds to the needs of young people.

For example the national network of young people living with HIV in Burundi (RNJ+) runs a youth centre which serves adolescents and young people living with HIV and from key populations [9]. The network has a clinic on site where young people both deliver and access HIV prevention, treatment and support services, such as counselling, health talks and HIV testing. The network has also integrated the use of information and communication technology to promote dialogue about sexual and reproductive health rights within the community and drive demand for services among young people.

While it is increasingly recognized that we are experts of our own lives [10], we must continue to push for greater investment in youth‐initiated interventions and youth–adult collaborations. We need to inspire transformation at scale so it becomes the norm to expect a youth‐led network or organization to be at the centre of large programmes for improving the health and wellbeing of adolescents and young people. The days of being on the side‐lines are over. We must be at the centre of these initiatives – and the examples shared show we can deliver if well‐resourced and mentored to do so.

Viva young people, viva!

## COMPETING INTERESTS

None of the authors have a competing interests.

## AUTHORS’ CONTRIBUTIONS

TR, AI, IN and SA contributed equally to this viewpoint. Each wrote sections of the initial draft, reviewed all revisions and approved the final version of the manuscript.

## AUTHOR INFORMATION

All authors are young HIV advocates and experts of their own lives. Tinashe G. Rufurwadzo is Communications, Evidence and Influence Advisor at the Global Network of Young People Living with HIV (Y + Global). Audrey Inarunkundo is Executive Director at Réseau National des Jeunes Vivant avec le VIH (RNJ+). Ikka Noviyanti is Advocacy Officer at Youth LEAD Asia Pacific Network of Young Key Populations. Miguel A. Subero is Regional Coordinator at the Regional Network of Youth living with HIV, Latin America and the Caribbean (J + LAC).

## ABBREVIATIONS

HIV, human immuno‐deficiency virus; ICT, information and communication technology; READY, Resilient Empowered Adolescents and Young People; RNJ+, Réseau National des Jeunes Vivant avec le VIH; SRHR, sexual and reproductive health and rights; UN, United Nations.
